# Spectrum of neuropsychological challenges in Turner syndrome

**DOI:** 10.3389/fendo.2024.1461103

**Published:** 2024-11-18

**Authors:** Jacqueline I. Fezza, Srishti Rau, Lauren Clary, Oretha Nimene Johnson, Fiona Fimmel, John Barber, Roopa Kanakatti Shankar

**Affiliations:** ^1^ The George Washington University School of Medicine and Health Sciences, Washington, DC, United States; ^2^ Division of Neuropsychology, Children’s National Hospital, Washington, DC, United States; ^3^ Division of Endocrinology, Children’s National Hospital, Washington, DC, United States; ^4^ Biostatistics, Children’s National Hospital, Washington, DC, United States

**Keywords:** Turner syndrome, neuropsychology, genetic syndromes, learning disorder, anxiety

## Abstract

**Introduction:**

Turner syndrome (TS) is associated with significant neuropsychological challenges, and screening is recommended at key transition stages. Our goal was to describe the institutional experience of formal neuropsychological assessments in TS and assess differences by karyotype.

**Methods:**

Data were abstracted by retrospective chart review of completed assessments between January 1, 2019, and October 31, 2022, referred from the newly established multidisciplinary clinic, and descriptive statistical analyses were presented (SAS V9.4).

**Results:**

Of 114 patients, 38 (33%) had completed neuropsychological assessment at a median age of 11.3 years (IQR 6.5–14.9). Median full-scale IQ (FSIQ) was lower in those with a 45,X karyotype compared with other karyotypes (p = 0.027) but did not meet statistical significance at the adjusted significance level for multiple comparisons. Lower median non-verbal IQ [performance intelligence quotient (PIQ)] relative to verbal IQ (VIQ) was observed. Diagnoses of attention-deficit hyperactivity disorder (ADHD) (26%) and anxiety disorder (26%) were common followed by specific learning disorder (mathematics; 18%) and autism spectrum disorder (16%).

**Discussion:**

The prevalence of neuropsychological abnormalities in our diverse clinic underscores the importance of early and routine neuropsychological testing in TS.

## Introduction

Turner syndrome (TS) is a genetic condition with a partial or completely missing second sex chromosome in a phenotypic female individual. Typical features include short stature, primary ovarian insufficiency, congenital heart defects, renal and skeletal anomalies, lymphedema, hearing impairment, metabolic syndrome, and neurocognitive difficulties (1). Approximately half of individuals with TS present with monosomy X, and the rest have varying karyotypes with significant variability in the phenotypic spectrum (1). There is also considerable variability in the neurocognitive profile, but some typical findings in TS include overall intellectual functioning in the average range, strong verbal abilities, and weaknesses in mathematics, executive function, visuospatial functioning, and processing speed (2, 3). Nearly 20%–30% may be diagnosed with a mental health (anxiety and depression) or neurodevelopmental disorder such as attention-deficit hyperactivity disorder (ADHD) and autism spectrum disorder (ASD) (4). In addition, individuals with TS demonstrate a higher risk of impairment in facial recognition, deciphering subtle social cues, fear recognition, and gaze processing (4, 5) and have been reported to have difficulties forming close relationships (6). Hence, the latest international consensus clinical guidelines recommend the integration of behavioral services into the care team for TS, routine screening for neurobehavioral concerns, and neuropsychological testing at key transition stages for TS patients (1).

The primary driver of neurocognitive impairment appears to be the haploinsufficiency of the genes on the X chromosome (7). Estrogen deficiency at critical stages of neurodevelopment perinatally as well as during puberty may impact neurocognition in TS (8). Early screening and intervention may improve the lives of individuals with TS and their families by identifying challenges and providing support to mitigate their impact. The goal of our study was to describe the institutional experience of establishing neuropsychological testing referrals and protocols as a component within a newly established multidisciplinary TS clinic to assess the prevalence of neuropsychological impairments and their clinical correlates (karyotype and age at the start of estrogen). We expected to see an increased prevalence of ADHD and anxiety as well as weakness in executive function and mathematics. We hypothesized that the prevalence of weaknesses in these domains (executive function, learning/memory, non-verbal reasoning, visual/spatial, and anxiety) may be higher in those with a 45,X karyotype compared to other karyotypes and correlate with a later age at estrogen start. We also hypothesized that we may find a positive association between weakness in domains of anxiety and executive functioning.

## Methods

A retrospective chart review (deemed exempt by the Institutional Review Board) was undertaken to review completed neuropsychological assessments between January 1, 2019, and October 31, 2022, on patients with TS referred from the newly established TS clinic in January 2019. All TS patients seen in the TS clinic were seen by a psychologist as part of the multidisciplinary clinic and referred for formal neuropsychological testing as part of routine care (e.g., the youth had not completed neuropsychology testing previously and the screening psychologist and/or family reported cognitive concerns). The majority of the neuropsychological assessments were completed by one of three neuropsychologists (SR, LK, or DP), with a few exceptions. Despite long wait lists, neuropsychologists reserved appointment slots for patients from the clinic, and referrals were usually scheduled within 6 months after submitting an intake form. The neuropsychological test battery was individualized to the patient’s age and presenting concerns. Neuropsychologists integrated test scores with the patient’s developmental history to provide an interpretation of their strengths and weaknesses diagnostic conceptualization (using ICD-10/DSM-5 nosology) and provide treatment recommendations, all of which were documented in an evaluation report. Since problems with executive functioning and anxiety were anticipated, the study team also abstracted data from the parent and self-reported “global executive composite” (GEC) score on the Behavior Rating Inventory of Executive Function [second edition, Behavior Rating Inventory of Executive Function (BRIEF-2) in the majority, but measures from BRIEF-1 were compiled where available instead] as well as the Internalizing, Externalizing, and Total Problems scores and Anxiety related scores on the Child Behavior Checklist (CBCL) instruments, which were administered to the majority of patients across different ages. The karyotype, age at TS diagnosis, age at thelarche and menarche and whether spontaneous/induced, age of estrogen initiation if applicable, birth weight, gestational age, and history of cardiac surgery were also abstracted from the medical record.

Descriptive statistics were used to summarize the data. Quantitative variables were expressed either with a mean and standard deviation or with a median and interquartile range depending on their underlying distribution. Categorical variables were expressed with their respective counts and frequencies. Statistical testing of the quantitative variables was performed using the Wilcoxon rank-sum test, and categorical variables were tested using the chi-square test or Fisher’s exact test depending on the counts with the Bonferroni correction for multiple comparisons. Further correlations between variables were also tested using Spearman’s rank correlation. All analyses were performed in SAS V9.4 (Cary, NC, USA) with two-sided tests, and a p < 0.05 was deemed statistically significant.

## Results

Of 114 unique patients (median age 9.55 years, IQR 5.7–14.7) seen in the TS clinic before August 31, 2022, 38 had completed neuropsychological assessment at a median age 11.3 years (IQR 6.5–14.9) by October 31, 2022. **Table 1** shows the comparison of age and karyotype of those who completed neuropsychological assessment compared with those seen in the TS clinic in the above time frame. The patients who completed neuropsychological assessments had a mean birth weight of 2.7 kg, and 8/38 (21%) had a history of cardiac surgery. At the time of the assessment, 19/38 (50%) were on growth hormone therapy, and 7/38 (18%) had previously completed growth hormone therapy. Of 19 pubertal patients, nine (47%) had spontaneous thelarche at a mean age of 12 years (SD 1.8), and 4/12 (33%) of these had spontaneous menarche at a mean age of 12.8 years (SD 0.6). The mean age at estrogen initiation was 13.2 years (SD 1.9 years).

**Table 1:** Demographics of patients with completed visits for neuropsychology tests compared to Turner syndrome clinic visits.

	Neuropsychological assessment completed N = 38	TS clinic visit N = 114
**Age in years at visit** Median (IQR)	11.3 (6.5–14.9)	9.55 (5.8–14.7)
Race (self-reported)		
Non-Hispanic White	17 (45%)	44 (38.5%)
Black/African American	7 (18.5%)	18 (16%)
Asian	1 (2.5%)	11 (9.5%)
Other	11 (29%)	35 (31%)
Unknown	2 (5%)	6 (5%)
Ethnicity (self-reported)		
Hispanic/Latinx	10 (26%)	30 (26%)
Age at diagnosis, N (%)		
1. Prenatal	7 (18%)	12 (11%)
2. Postnatal	31 (82%)	78 (89%)
If postnatal, age in years Median (IQR)	4.0 (0.8–11.0)	5.3 (0.9–10)
Karyotypes N (%)		
1. 45,X (monosomy)	15 (39%)	47 (41%)
2. Ring X	2 (5%)	13 (11%)
3. Isochromosome Xq	11 (29%)	20 (18%)
4. Presence of Y-chromosome	4 (10.5%)	8 (7%)
5. 45,X/46,XX	1 (3%)	9 (8%)
6. 45,X/47,XXX	1 (3%)	2 (2%)
7. Other	4 (10.5%)	15 (13%)

Neuropsychological results were stratified by 45,X vs. other karyotypes. Median full-scale intelligence quotient (FSIQ) was significantly lower in those with a 45,X karyotype (median FSIQ 76, IQR 51–88) compared with other karyotypes (median FSIQ 95, IQR 75–103) (p = 0.027) but did not reach statistical significance when the Bonferroni correction was applied for multiple comparisons (statistical significance at p < 0.004). We observed a trend of lower median non-verbal (performance) IQ (PIQ) relative to verbal IQ (VIQ) in both groups [45,X: median (IQR) for VIQ 84 (72–108) and PIQ 79 (63–91); other karyotypes: VIQ 102 (85–113) and PIQ 88 (73–97)].

Parent-reported T-scores (mean 50, SD 10) on BRIEF-2 (including a few BRIEF-1 scores) and CBCL are shown in **Table 2**. The median T-scores were within one SD and did not differ by 45,X karyotype compared with others. However, several patients had clinically elevated scores across the measured domains as depicted in **Table 2**. For available dyads (N = 12), the parent and self-reported GEC scores were positively correlated (Rho = 0.775, p = 0.003).

**Table 2:** Parent-reported T-scores (mean 50, SD 10) on Behavior Rating Inventory of Executive Function, second edition (BRIEF-2) and Child Behavior Checklist (CBCL).

Scores	Median T-scores	Interquartile range	N (%) clinically elevated, (definition)
BRIEF Global Executive Composite (GEC) (N = 33)	58	45–64	3/33 (9%), (>70)
CBCL Total Problems (N = 34)	55	44.5–63	7/34 (21%), (>63)
CBCL Internalizing Problems (N = 33)	56	47–64	9/33 (27%), (>63)
CBCL Externalizing Problems (N = 33)	52	41–60	5/33 (15%), (>63)
CBCL Anxious/Depressed (N = 33)	56	51–63	2/33 (6%), (≥70)
CBCL DSM-5 Anxiety Problems (N = 33)	58	51–67	6/33 (18%), (≥70)

**Figure 1:**
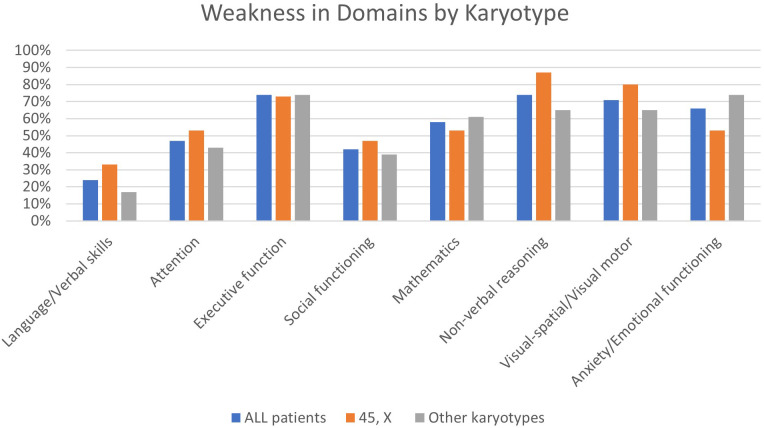
Weakness in domains by karyotype in Turner syndrome.

**Figure 2:**
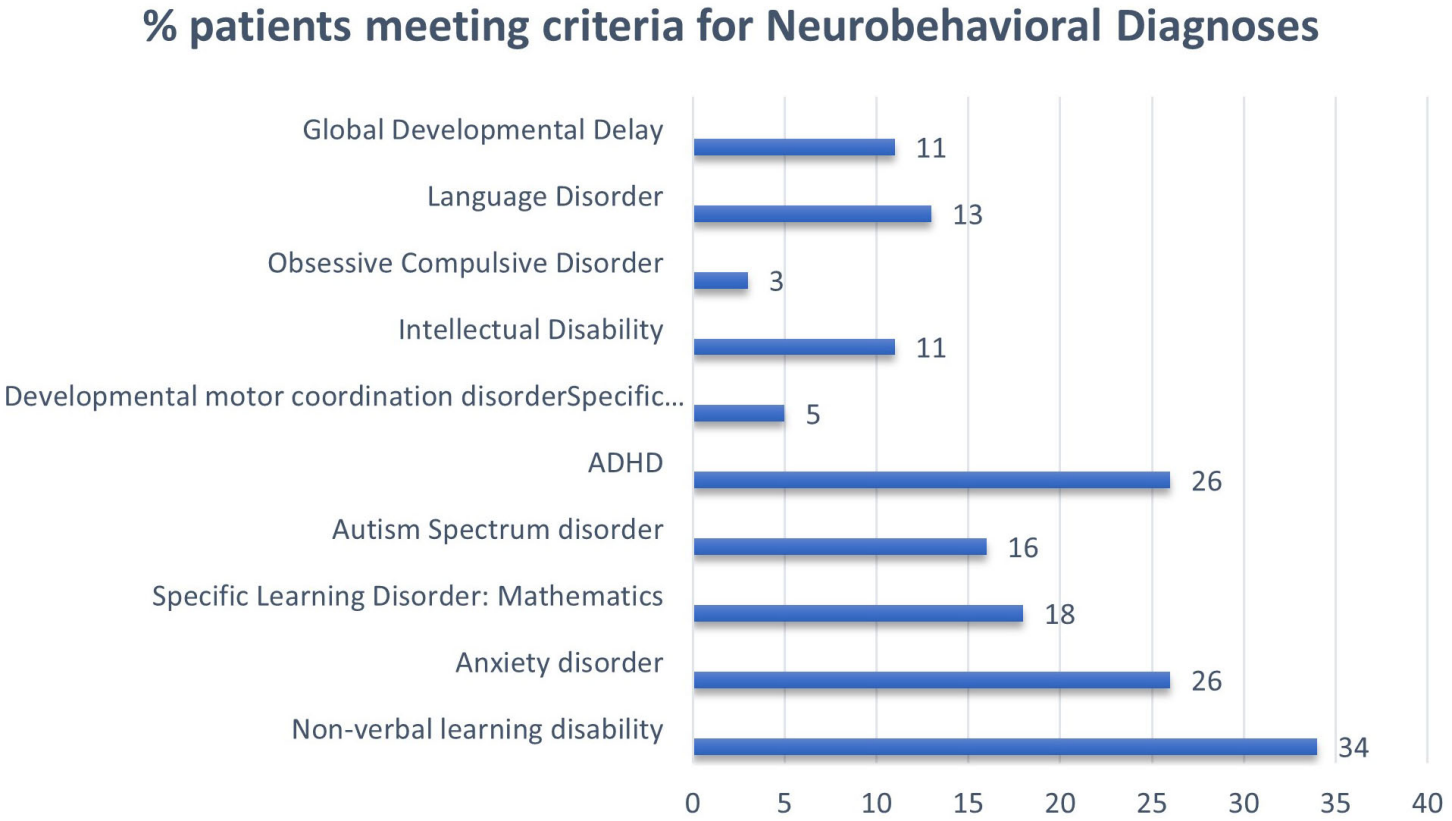
Prevalence of neurodevelopmental and behavioral diagnoses on formal evaluation in patients with Turner syndrome.

**Figure 1** shows the prevalence of weaknesses (as interpreted by neuropsychologists) in several domains for patients in our institutional sample. Weaknesses in visuospatial/visual-motor functioning, non-verbal reasoning, mathematics, and executive function as well as anxiety were present in over 50% of individuals tested. A non-verbal learning disability profile was evident in approximately one-third (34%) of the patients. Results were stratified by 45,X vs. other karyotypes, although there were no statistically significant differences between the groups. **Figure 2** shows the prevalence of neurobehavioral diagnoses meeting DSM-5 criteria in this cohort. Diagnoses of ADHD (26%) and anxiety disorder (26%) were common followed by specific learning disorders (mathematics; 18%) and ASD (16%).

A positive correlation was found between weakness in the anxiety domain and weakness in executive function (p = 0.016) as hypothesized *a priori*. We examined the correlation between age at estrogen treatment start and weakness in domains (executive functioning, learning/memory, non-verbal reasoning, visual/spatial, and anxiety) and did not find any statistically significant relationships.

## Discussion

Our study is a retrospective chart review of neuropsychological evaluation reports and medical records for patients with TS in a newly established multidisciplinary TS clinic at our institution. Despite a streamlined approach to referrals and sooner appointment slots, only one-third of patients had completed formal neuropsychological assessments in that time frame. We found a high prevalence of anxiety, difficulties in math, non-verbal learning disability profile, and executive functioning as anticipated. In addition, the mean full-scale IQ in our sample was 94.6, which was comparable (i.e., also in the average range) to that of a separate cohort of female individuals with TS (FSIQ = 103.9) (9). In our sample, patients with a 45,X karyotype had lower median FSIQ (76) compared with other karyotypes (95), which is a novel finding but needs to be verified in larger cohorts of patients with TS. The finding was no longer significant after the application of the Bonferroni correction for multiple comparisons, but a significant result may be missed with this approach due to the risk of false negatives (type II error) with the small sample size. Some prior studies have also suggested lower IQ in those with 45,X compared with mosaic TS patients (10), but other studies have not reported an impact of karyotype on IQ (11). There were also demonstrable differences in gray matter development in those with 45,X karyotype compared to patients with other TS karyotypes (11), but genetic underpinnings are yet to be proven (2). It remains unclear why 45,X is associated with a more severe neurocognitive phenotype and may potentially be related to genetic haploinsufficiency for specific genes that impact gray matter development and maturation. However, 45,X is also associated with a higher incidence of ovarian insufficiency and estrogen deficiency, and the early hormonal milieu may also play a role in neurocognitive development. The lower FSIQ in patients with a 45,X karyotype, is driven primarily by a lower non-verbal IQ (PIQ), and this split pattern of lower PIQ compared to VIQ has been consistently recognized in TS (1, 12) and underscores the importance of recognizing the imbalance in cognitive capabilities of individuals with TS (as opposed to indicating broad cognitive impairment) and the need to leverage individual strengths to address their potential challenges. Our finding of intellectual disability in 11% of individuals is consistent with prior reports (1).

Our data confirm the high prevalence of neuropsychological impairment among patients with TS in our clinic, and the findings are in line with previous reports, highlighting weaknesses in visuospatial ability, mathematics, and executive function and challenges with anxiety in youth with TS (1, 4). Overall, the high prevalence of neurocognitive difficulties justifies the need for routine neuropsychological screening and, if indicated, further assessment by trained clinicians. Such assessment is particularly important considering the observation of relative underestimation of TS patients’ executive function- and anxiety-related difficulties by parents (i.e., median BRIEF-2 and CBCL scores broadly within normal ranges) despite considerable proportions of patients meeting criteria for attention and anxiety disorders following neuropsychological assessment (**Table 2**). Approximately 26% met the criteria for diagnosis of ADHD in our cohort similar to prior reports (5), higher than the estimated 8% in the general population of girls in the USA (13). ASD was also formally diagnosed in 16% of our cohort, while previous reports have been variable ranging from estimates of 3% up to 23% (14–16). Over 25% also met the DSM-5 criteria for an anxiety disorder comparable to previous reports of 11%–30% (17) compared to 9% in the general population (18). A community-based mixed methods study confirmed that anxiety is often underdiagnosed in childhood and suggested that anxiety triggers appear to be related to problems associated with TS (reduced social cognition, executive function deficits, and health-related concerns due to infertility or cardiovascular disease) (19). Anxiety impacts the whole family, and early signs of anxiety are often misinterpreted, with missed opportunities for early identification and intervention (19). Although the association of weakness in executive function and anxiety that we noticed in our retrospective study does not imply causation, the potential nature of this association needs further exploration.

We recognize several limitations inherent in a retrospective single institutional study, especially the small sample size, which may affect the precision of our estimates and the generalizability of the results. Another limitation is a potentially biased sample due to delayed assessments during the COVID-19 pandemic; completed assessments may reflect patients with more significant impairment considering parents of more impacted patients are more motivated and likely to seek/follow-up on a referral for assessment. The selection bias may have skewed our estimates to a higher prevalence and severity of cognitive deficits. Unrecognized confounders in our patient sample referred to a tertiary care center may also explain some of the findings. The lack of a control group also makes it difficult to determine whether all of the observed cognitive differences are attributable to TS. However, the prevalence estimates in our study match prior studies and have all been shown to be consistently higher than the prevalence in the general population.

At the same time, our clinic represents an ethnically diverse population in a rare disease that mirrors real-world data and reflects the inherent challenges that families face when trying to complete formal neuropsychological assessment, despite the support of a multidisciplinary team prioritizing these referral visits. It also highlights the value and need for formal assessments as the standard of care to ensure timely recognition of neuropsychological impairments to ensure that supports are established (e.g., academic and social–emotional support and monitoring at school and additional therapies) in a timely manner to improve the quality of life of individuals and their families. Knowledge of neuropsychological strengths and challenges of young adult patients with TS will help healthcare providers adequately address their concerns and prepare them for the transition to adult care (21). Recognition of ADHD, anxiety, and other neuropsychological challenges experienced by individuals with TS is like the proverbial tip of the iceberg and has far-reaching impacts on individuals’ academic achievements, social connections, and emotional wellbeing. Even when identified, the ability to get adequate intervention and support in the academic setting or mental health resources in the community is yet another challenge to surmount. The need for evidence-based interventions is recognized in a recent survey of research priorities for the TS community: individuals and their families picked anxiety disorders, social deficits, and learning disabilities as well as quality of life and transition of care among the top health priorities on par with fertility and hormone replacement concerns (20).

Future studies will need to explore barriers to completion of neuropsychological assessments, optimal assessment tools specific to this population, testing frequency, and the impact of test results on treatment planning. Large multicenter studies with prospective designs and studies of diverse patient populations and resource settings that capture socio-economic and linguistic cultural determinants are essential to comprehend the extent of neuropsychological challenges in this community. It is also important to partner with the TS community to address the felt needs and establish evidence of interventions on quality of life. It remains to be seen if early recognition and academic support for some of these challenges such as executive function and ADHD may indeed improve anxiety symptoms and self-esteem. Another area of study is the impact of timely estrogen hormone replacement during puberty and whether it may help improve performance on neurocognitive measures. Comparative effectiveness studies on time, dose, and formulation of estrogen/progesterone on the neurocognitive outcomes are imperative.

In conclusion, our findings demonstrate a broad spectrum of neuropsychological challenges in patients who completed neuropsychological assessments referred from a multidisciplinary Turner syndrome clinic. Our study begins to examine the karyotypic differences and clinical correlates of impairments in neurocognitive domains. We found that youth with 45,X had a lower median full-scale IQ compared with other karyotypes and confirmed a higher verbal IQ compared with non-verbal/performance IQ. Although lower than average, the FSIQ in TS remains broadly within the “normal range”, and it is important to recognize the strengths in verbal IQ. A high prevalence of weaknesses in non-verbal reasoning, mathematics, and executive function as well as diagnoses of ADHD, ASD, and anxiety disorder are also confirmed in our cohort. Further study in larger cohorts of optimal treatment and multidisciplinary support that is tailored to the unique neuropsychological challenges in TS is needed.

## Data Availability

The deidentified dataset is not publicly shared due to this being a rare disease and possibility of patients able to be identified but is available on enquiry from the corresponding author.
